# The $\chi$-Binding Function of *d*-Directional Segment Graphs

**DOI:** 10.1007/s00454-025-00737-2

**Published:** 2025-05-17

**Authors:** Lech Duraj, Ross J. Kang, Hoang La, Jonathan Narboni, Filip Pokrývka, Clément Rambaud, Amadeus Reinald

**Affiliations:** 1 Theoretical Computer Science Department, Faculty of Mathematics and Computer Science, Jagiellonian University, Krakow, Poland; 2 Korteweg-de Vries Institute for Mathematics, University of Amsterdam, Amsterdam, Netherlands; 3 Faculty of Informatics, Masaryk University, Brno, Czech Republic; 4 Université Côte d’Azur, CNRS, Inria, I3S, Sophia-Antipolis, France; 5 LIRMM, Université de Montpellier, CNRS, Montpellier, France

**Keywords:** Geometric graphs, Chi-boundedness, Segment graphs, Graph colouring

## Abstract

Given a positive integer *d*, the class *d*-DIR is defined as all those intersection graphs formed from a finite collection of line segments in ${\mathbb R}^2$ having at most *d* slopes. Since each slope induces an interval graph, it easily follows for every *G* in *d*-DIR with clique number at most $\omega$ that the chromatic number $\chi (G)$ of *G* is at most $d\omega$. We show for every even value of $\omega$ how to construct a graph in *d*-DIR that meets this bound exactly. This partially confirms a conjecture of Bhattacharya, Dvořák and Noorizadeh. Furthermore, we show that the $\chi$-binding function of *d*-DIR is $\omega \mapsto d\omega$ for $\omega$ even and $\omega \mapsto d(\omega -1)+1$ for $\omega$ odd. This extends an earlier result by Kostochka and Nešetřil, which treated the special case $d=2$.

## Introduction

In structural graph theory, a fundamental task is to characterise the complex, global parameter of chromatic number $\chi$ in terms of the simpler, more local parameter of clique number $\omega$. For a given graph class, the question of whether this task is even well-defined belongs to the theory of $\chi$-boundedness, an area systematically initiated by Gyárfás in the 1980s [5]. A graph class $\mathcal {G}$ is called $\chi$*-bounded* if there is some function $f_\mathcal {G}$ such that $\chi (G) \le f_\mathcal {G}(\omega (G))$ for any $G\in \mathcal {G}$, and, if that is so, the optimal choice of $f_\mathcal {G}$ is called the $\chi$*-binding* function of $\mathcal {G}$. Note that the triangle-free graphs of arbitrarily large chromatic number give rise to many interesting graph classes that are not $\chi$-bounded. This area of mathematics is deep and active, but despite many recent advances, there are many important classes for which the question of $\chi$-boundedness is difficult and remains open; for a nice recent account of the state of the art, see [11]. Suffice it to say that determination of the $\chi$-binding function for a nontrivial (non-perfect) class is generally considered a rarity.

Since even before Gyárfás’s early work (see [1]), much attention has been focused on intersection classes. These are graph classes defined by taking some natural collection of sets, usually geometrically-defined, and forming for every finite subcollection of those sets an auxiliary graph in which each vertex corresponds to a set, and two vertices are connected by an edge if and only if the corresponding sets have a nontrivial intersection. Such a restriction of focus is not too confining, not only because many intersection classes are fundamental to the structural understanding of graphs (consult, e.g. [9, 12] for more context), but also because intersection classes are more than rich enough for its $\chi$-boundedness theory to contain many fascinating challenges.

As one prominent (and pertinent) example, consider the collection of straight line segments in the plane $\mathbb {R}^2$ (or more formally, the collection of those closed intervals drawn between some pairs of points in $\mathbb {R}^2$). Its intersection class is called the *segment (intersection) graphs*. If all the segments happen to lie in parallel, then the resulting segment graph is an interval graph, and thus perfect; that is, it has equality between $\chi$ and $\omega$. One should not expect this to remain true when we allow the segment slopes to vary, but it is reasonable to ask if there might remain a good relationship between $\chi$ and $\omega$, that is, are segment graphs $\chi$-bounded? Indeed, Erdős asked this in the 1970s (see [5, 10] for more details of the problem’s provenance).

Despite sustained attention and nice partial results (see e.g. [6, 8, 13]), Erdős’s innocent-looking but very difficult challenge was not resolved until 2014, and in the negative. Work of Pawlik et al. [10] provided a strikingly elegant construction of triangle-free segment graphs of arbitrarily large chromatic number. Interestingly, the graphs produced by the construction are isomorphic to the intersection graphs that were exhibited by Burling [3] to show that the intersection class for axis-aligned boxes in ${\mathbb R}^3$ is not $\chi$-bounded.

One might consider this a coda, but permit us to prolong the narrative, in particular by parameterising according to the number of segment slopes. We first note that the construction of Pawlik et al. must have arbitrarily many slopes. Suppose to the contrary that the segments admit at most *d* slopes. By the same observation as above, each slope induces a perfect graph *H* having $\chi (H)$ equal to $\omega (H)\le 2$. By colouring the slopes disjointly from one another, we can conclude that $\chi$ is at most 2*d* for the entire graph, a contradiction.

More generally, given a positive integer *d*, we refer to the *d-directional segment graphs* as those intersection graphs formed from a collection of line segments in $\mathbb {R}^2$ having at most *d* distinct slopes. Call the class of such graphs *d-DIR*. (Take note of the subtlety that this is not *per se* an intersection class, but rather a union of many of them.) What we just argued is that $\chi (G) \le d\omega (G)$ for any *G* in *d*-DIR. Thus, in a simple fashion, we can conclude that the graph class *d*-DIR is $\chi$-bounded.

The question we would like to address here is, what is the $\chi$-binding function of *d*-DIR? This question was raised first around the turn of the century by Kostochka and Nešetřil [7] and again more recently by Bhattacharya et al. [2]. In both works, it was proved that there are 2-directional segment graphs of clique number 2*t* and chromatic number 4*t*, for all $t\in \mathbb {N}$, which attains the bound of the above simple argument in this special case. Bhattacharya et al. moreover conjectured that that simple bound is optimal in general, that is, that $\omega \mapsto d \omega$ is the $\chi$-binding function of *d*-DIR for all $d>2$.

We succeeded in completely resolving this question as follows.

### Theorem 1

The $\chi$-binding function of *d*-DIR is

$$\begin{aligned} \omega \mapsto {\left\{ \begin{array}{ll} d\omega &  \text {if } \omega \text { is even, or}\\ d(\omega -1)+1 &  \text {if } \omega \text { is odd.} \end{array}\right. } \end{aligned}$$

One can interpret this statement as a graceful generalisation of perfection in the $d=1$ case. We note that Kostochka and Nešetřil [7] had already established the $d=2$ case of Theorem 1, unbeknownst to the authors in [2]. This had already refuted the conjecture in [2] in this special case.

The crux in Theorem 1 is to prove the lower bound in the even $\omega$ case. Here is a rough sketch of that construction, which at a high level combines aspects of both the construction of Pawlik et al. and that of Bhattacharya et al. and indeed strengthens/refines both constructions. We start by fixing $\omega =2$ and proceed by induction on *d*, exhibiting triangle-free graphs with *t*-fold chromatic number at least 2*td*. We refine the induction used by Pawlik et al. by controlling, at every step, the growth of the number of slopes required for achieving a given *t*-fold chromatic number. We then obtain the result for all even $\omega >2$ by blowing up each segment by a factor $\omega /2$.

The structure of the paper is as follows. In Sect. 2, we lay the ground for our proof by introducing the notions and tools used throughout the paper. Then we settle the even $\omega$ case in Sect. 3. The lower bound for the odd $\omega$ case in Sect. 4 is a mild adaptation of the even $\omega$ construction. We give a short proof of the upper bound for the odd $\omega$ case in Sect. 5. We end with some open questions for future research in Sect. 6.

## Preliminaries

### *t*-Fold Colourings

For all positive integers *a* and *t*, a *t-fold a-colouring* of a graph *G* is a function $$\phi :V(G) \rightarrow \left( {\begin{array}{c}\{1,\dots ,a\}\\ t\end{array}}\right) $$ such that for every edge $uv \in E(G)$, $\phi (u) \cap \phi (v) = \emptyset$. For $t=1$, we will often refer to $\phi$ simply as an *a-colouring*. For any *t*-fold colouring $\varphi$ of a graph *G*, if *X* is a set of vertices of *G*, we denote by $\varphi (X)$ the set of colours used to colour the vertices in *X*, i.e. $\varphi (X) = \bigcup _{v\in X}\varphi (v)$. We will also use the notation $\varphi _{\vert X}$ to denote the restriction of the function $\varphi$ to the set *X*.

### Segments

A *segment* *S* with endpoints $A=(x_1,y_1)$ and $B=(x_2,y_2)$ in $\mathbb {R}^2$ is the set $\{A+\lambda (B-A) \mid \lambda \in [0,1]\}$. The *length of a segment* is its Euclidean norm. A *rectangle* *R* is defined as the Cartesian product of two segments $[a,c] \times [b,d]$ with $a,b,c,d \in \mathbb {R}$ and $c>a$ and $d>b$. A rectangle always has non-zero area. If the two intervals have the same length, we say that *R* is a *square*. We naturally define the *left*, *right*, *top* and *bottom* *sides* of a rectangle *R* as the four segments whose union is the boundary of *R*. The left and right side of *R* are the vertical sides of *R*, and the top and bottom sides of *R* are the horizontal sides of *R*. The length of a vertical side is the *height* of *R*, the length of a horizontal side is the *width* of *R*. The aspect ratio of *R* is the height divided by the width of *R*. A segment *S* *crosses a rectangle R vertically or horizontally* if *S* intersects the two horizontal or vertical sides of *R*, respectively. We say that a rectangle $R_1$ crosses a rectangle $R_2$ vertically (respectively, horizontally) if the left and right sides (respectively, the top and bottom sides) of $R_1$ cross the rectangle $R_2$ vertically (respectively, horizontally). If $R_1$ crosses $R_2$ vertically, then $R_2$ crosses $R_1$ horizontally. The *slope* of a segment in $\mathbb {R}^2$ with endpoints $(x_1,y_1),(x_2,y_2)$ is $\frac{y_2-y_1}{x_2-x_1}$ if $x_1\ne x_2$, and $\infty$ otherwise. The *slope number* of a finite family $\mathcal {S}$ of segments is the cardinality of the set of the slopes of segments in $\mathcal {S}$.

Given a set $\mathcal {S}$ of segments, the *intersection graph* $G(\mathcal {S})$ of $\mathcal {S}$ is the graph with vertex set $\mathcal {S}$ and with edges consisting of all pairs $SS'$ with $S, S' \in \mathcal {S}$ such that $S \cap S' \ne \emptyset$.

A *t-blowup of a segment* is a multiset of *t* copies of the same segment. A *t-blowup of a set of segments* is the multiset of the *t*-blowup of the segments. A *t-blowup of a graph* is the graph obtained from *G* by replacing every vertex by a copy of $K_t$, and every edge by a copy of $K_{t,t}$. Observe that a *t*-fold colouring of *G* corresponds to a colouring of the *t*-blowup of *G*.

### Configurations

We now introduce the notion of probes, first used in [10]. Let $\mathcal {S}$ be a family of segments in *the unit square* $\mathcal {U}= [0,1] \times [0,1]$. Let $P = [a,c] \times [b,d]$ be a rectangle included in $\mathcal {U}$. We denote by $\mathcal {S}(P)$ the set of segments in $\mathcal {S}$ intersecting *P*. The *right-extension* of *P* is the rectangle $[a,1] \times [b,d]$. The rectangle $P = [a,c] \times [b,d]$ is a *probe* for $\mathcal {S}$ if the following hold:

- $c=1$, that is, the right side of *P* lies on the boundary of $\mathcal {U}$;
- all segments in $\mathcal {S}(P)$ cross *P* vertically; and
- all segments in $\mathcal {S}(P)$ are pairwise disjoint.

Given a probe *P*, a *root* of *P* is a rectangle $[a,c^{\prime }] \times [b,d]$ where $c^{\prime }$ is a real number such that $[a,c^{\prime }] \times [b,d]$ is disjoint from every segment in $\mathcal {S}$.

A *configuration* is a pair $\mathcal {C} = (\mathcal {S},\mathcal {P})$ with $\mathcal {S}$ a set of segments in $\mathcal {U}$ and $\mathcal {P}$ a family of pairwise disjoint probes for $\mathcal {S}$.

For convenience, we define the intersection graph $G(\mathcal {C})$ of a configuration $\mathcal {C}=(\mathcal {S},\mathcal {P})$ as $G(\mathcal {S})$. We say that $\mathcal {C}$ is triangle-free if $G(\mathcal {S})$ is a triangle-free graph.

Let *R* be a square inside $\mathcal {U}$ and let $\psi$ be the positive homothety mapping $\mathcal {U}$ to *R*. The *R-scaled copy* of $\mathcal {C}$ is the configuration $\mathcal {C}' = (\mathcal {S}',\mathcal {P}')$ where $\mathcal {S}'$ is the set of images of the segments in $\mathcal {S}$ by $\psi$ and $\mathcal {P}'$ is the set of the right-extensions of the images by $\psi$ of the probes in $\mathcal {P}$. Observe that $\mathcal {S}'$ has the same set of slopes as $\mathcal {S}$.

### Copying Configurations

Our refinement on the construction of [10] is obtained at the cost of adding significantly many more copies of the construction achieving $\chi$ colours when building the graph requiring $\chi +1$ colours. Here, we describe this operation. For every configuration $\mathcal {C}$ and for every positive integer *k*, we define the new configuration $\mathcal {C}^{(k)}$ with segments set $\mathcal {S}^{(k)}$ and probes set $\mathcal {P}^{(k)}$ as “a way of joining *k* independent copies of $\mathcal {C}$ together”. With every probe $P \in \mathcal {P}^{(k)}$, we associate a set of *k* sub-rectangles of *P* that we call the *pillars* of *P*. The pillars have the same height as *P*, are pairwise disjoint, and no pillar starts from the left side of *P* so that there is a root of *P* not belonging to any pillar. Informally, each pillar will correspond to a copy of $\mathcal {C}$.

Formally, $\mathcal {C}^{(k)}=(\mathcal {S}^{(k)},\mathcal {P}^{(k)})$ is defined as follows:

- If $k=1$, then $\mathcal {C}^{(1)} = \mathcal {C}$. For every probe *P*, we define its only pillar $I_0^P$ as the minimal rectangle containing *P* minus a root. Every segment of $\mathcal {S}(P)$ is a segment of $\mathcal {S}(I_0^P)$ and it also crosses $I_0^P$ vertically.
- If $k\ge 2$, then for every probe $P \in \mathcal {P}^{(k-1)}$ we choose a square $R_P$ in a root of *P* disjoint from its pillars and insert an $R_P$-scaled copy $\mathcal {C}_P = (\mathcal {S}_P,\mathcal {P}_P)$ of $\mathcal {C}$. We define $\mathcal {S}^{(k)} = \mathcal {S}^{(k-1)} \cup \bigcup _{P \in \mathcal {P}^{(k-1)}} \mathcal {S}_P$ and $\mathcal {P}^{(k)} = \bigcup _{P \in \mathcal {P}^{(k-1)}} \mathcal {P}_P$. In other words, instead of *P*, we take new probes, generated in $R_P$ by the new copy of $\mathcal {C}$, extended to the right. These new probes are then wholly contained in *P*. For every such probe $P'$, we define *k* different pillars of $P'$. There are $k-1$ pillars $I_0^{P'},I_1^{P'},\dots ,I_{k-2}^{P'}$ which are intersections between the pairwise disjoint pillars $I_0^{P},I_1^{P},\dots ,I_{k-2}^{P}$ of *P* and $P'$ and one pillar $I_{k-1}^{P'}$ that is the minimal rectangle containing $R_P \cap P'$ minus a root of $P'$. Since $R_P$ is contained in a root of *P*, $I_{k-1}^{P'}$ is also disjoint from $I_0^{P'},\dots ,I_{k-2}^{P'}$. Furthermore, for every $i\in \{0,\dots ,k-2\}$, since segments in $\mathcal {S}^{(k-1)}(I_i^P)$ cross $I_i^P$ vertically, they also cross $I_i^{P'}$ vertically. Therefore, $\mathcal {S}^{(k-1)}(I_i^P)\subseteq \mathcal {S}^{(k)}(I_i^{P'})$.

**Fig. 1:**
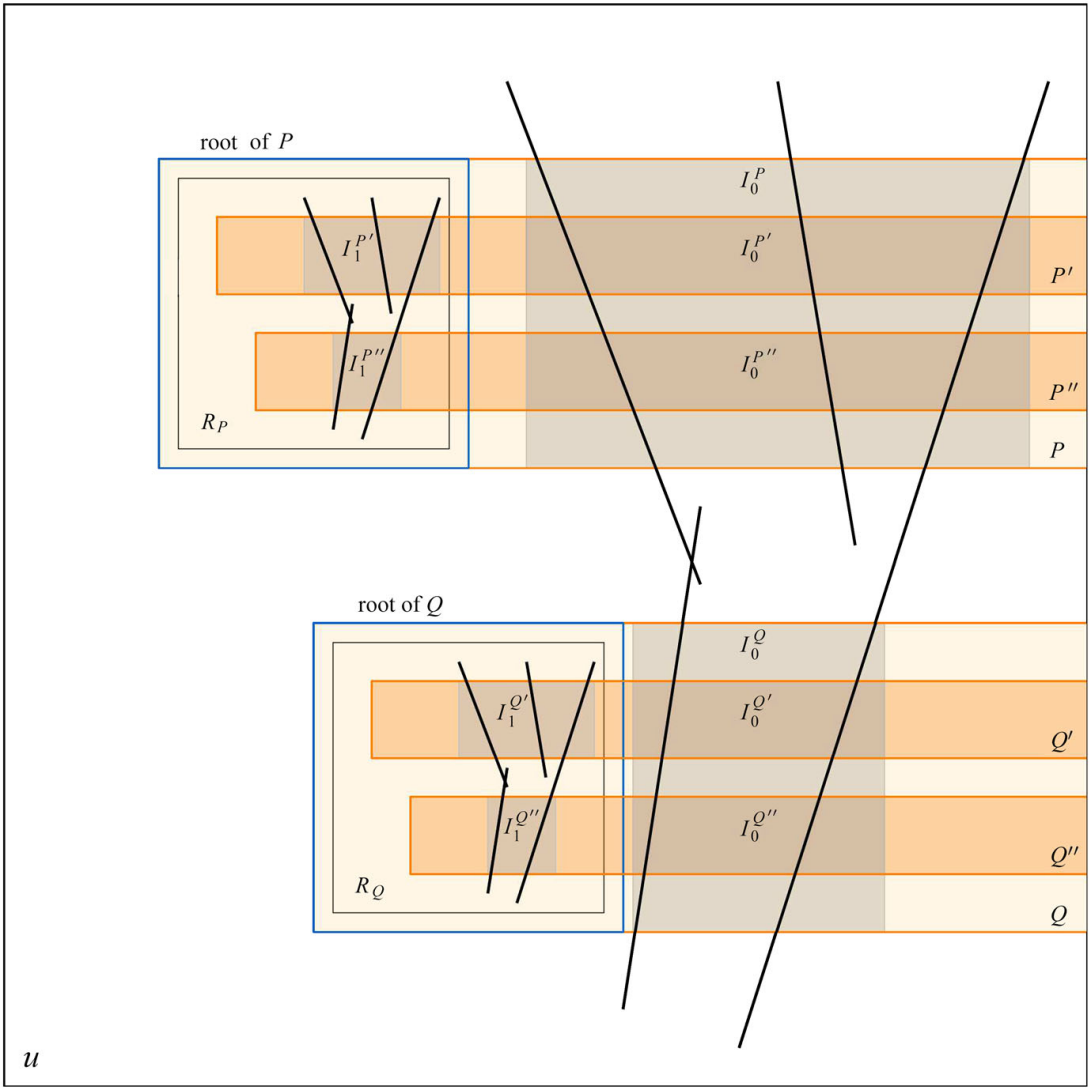
Building $$\mathcal {C}^{(2)}$$ from $$\mathcal {C}^{(1)}=\mathcal {C}$$

Note that $G(\mathcal {C}^{(k)})$ is the union of disjoint copies of $G(\mathcal {C})$, and in particular $\mathcal {C}^{(k)}$ is triangle-free if $\mathcal {C}$ is triangle-free. See Fig. 1.

#### Lemma 2

Let $\mathcal {C}=(\mathcal {S},\mathcal {P})$ be a configuration and let *t* be a positive integer. If there exists a nonnegative integer *s* such that, for every *t*-fold colouring $\phi$ of $G(\mathcal {C})$, there exists a probe $P\in \mathcal {P}$ such that $|\phi (\mathcal {S}(P))|\ge s$, then, for every *t*-fold colouring $\phi$ of $G(\mathcal {C}^{(k)})$, there exists a probe $P'$ such that, for every pillar *I* of $P'$, $|\phi (\mathcal {S}(I))|\ge s$.

#### Proof

The proof is an induction on *k*.

Suppose that $k=1$. Observe that $\mathcal {C}^{(1)}=\mathcal {C}$. Let $\phi$ be a *t*-fold colouring of $G(\mathcal {C}^{(1)})$. There exists a probe *P* such that $\big |\phi (\mathcal {S}(P))\big |\ge s$ by assumption on $\mathcal {C}$. Since every segment of $\mathcal {S}(P)$ crosses the only pillar *I* of *P* vertically, $\big |\phi (\mathcal {S}(I))\big |\ge s$. Therefore, $P'=P$ satisfies the conclusion of Lemma 2.

Suppose that $k\ge 2$. Let $\phi$ be a *t*-fold colouring of $G(\mathcal {C}^{(k)})$. By the inductive hypothesis, there exists a probe *P* of $\mathcal {C}^{(k-1)}$ such that for every $i\in \{0,\dots ,k-2\}$, every pillar $I^P_i$ of *P* satisfies $\big |\,\phi _{\vert \mathcal {S}^{(k-1)}}(\mathcal {S}^{(k-1)}(I_i^P))\,\big |\ge s$. By the definition of $\mathcal {C}^{(k)}$, there exists a square $R_P$ in a root of *P* with an $R_P$-scaled copy $\mathcal {C}_P=(\mathcal {S}_P,\mathcal {P}_P)$ of $\mathcal {C}$. By assumption on $\mathcal {C}$, there exists a probe $P'\in \mathcal {P}_P \subseteq \mathcal {P}^{(k)}$ such that $\big |\,\phi _{\vert \mathcal {S}_P}(\mathcal {S}_P(P'))\,\big |\ge s$. Since every segment of $\mathcal {S}_P(P')$ crosses $I^{P'}_{k-1}$ vertically, $\big |\,\phi (\mathcal {S}^{(k)}(I^{P'}_{k-1}))\,\big |\ge \big |\,\phi _{\vert \mathcal {S}_P}(\mathcal {S}_P(I^{P'}_{k-1}))\,\big | \ge \big |\,\phi _{\vert \mathcal {S}_P}(\mathcal {S}_P(P'))\,\big |\ge s$. For every $i\in \{0,\dots ,k-2\}$, every segment of $\mathcal {S}^{(k-1)}(I_i^P)\subseteq \mathcal {S}^{(k)}(I_i^P)$ crosses the pillar $I^{P'}_i$ vertically. Therefore, we also have $\big |\,\phi (\mathcal {S}^{(k)}(I^{P'}_i))\,\big | \ge \big |\,\phi _{\vert \mathcal {S}^{(k-1)}}(\mathcal {S}^{(k-1)}(I_i^P))\,\big |\ge s$. $\square$

## Lower Bound for Even $\omega$

In this section, we show the lower bound for the $\chi$-binding function of *d*-DIR for even clique numbers. First, we exhibit, for any *t* and *d*, a triangle-free *d*-DIR configuration with *t*-fold chromatic number at least 2*td*. Then, a *t*-blowup of this construction yields one with clique number 2*t* and chromatic number at least 2*td*, establishing the even case in Theorem 1.

### Theorem 3

For all positive integers *t* and *d*, there exists a triangle-free configuration $\mathcal {C}_{t,d} = (\mathcal {S}_{t,d}, \mathcal {P}_{t,d})$ with slope number at most *d* such that for every *t*-fold colouring $\phi$ of $G(\mathcal {C}_{t,d})$, there exists a probe $P \in \mathcal {P}_{t,d}$ such that $|\phi (\mathcal {S}_{t,d}(P))| \ge 2td$.

### Proof

We proceed by induction on *d*. For $d=0$, the configuration $(\emptyset , \{\mathcal {U}\})$ satisfies the statement of the theorem.

Suppose that $d>0$ and that $\mathcal {C}_{t,d-1}$ is already constructed. Let $\mathcal {H} = \mathcal {C}_{t,d-1}^{(4t+1)}=(\mathcal {S}_\mathcal {H},\mathcal {P}_\mathcal {H})$ be the copy of $4t+1$ of these configurations as defined in Sect. 2.4, which is triangle-free. Let $\Gamma$ be the set of the slopes of the segments in $\mathcal {S}_{t,d-1}$. We define the slope $\gamma$ as the minimum aspect ratio over all probes of $\mathcal {P}_\mathcal {H}$, divided by 4*t*.

We will construct a family of triangle-free configurations $\mathcal {H}_i = (\mathcal {S}_{i},\mathcal {P}_{i})$ for all $i \ge 0$ such that

1. for every segment $S \in \mathcal {S}_{i}$, the slope of *S* is in $\Gamma \cup \{\gamma \}$, and
2. for every *t*-fold colouring $\phi$ of $G(\mathcal {H}_{i})$, there is a probe $P \in \mathcal {P}_i$ such that $|\phi (\mathcal {S}_i(P))| \ge 2t(d-1)+2t(1-\frac{1}{2^{i}})$.

For $i = \left\lfloor \log (2t) \right\rfloor +1$, Item 2 implies that $|\phi (\mathcal {S}_i(P))| \ge 2td$, and so it suffices to take $\mathcal {C}_{t,d} = \mathcal {H}_i$.

We build $\mathcal {H}_i$ as follows. For $i=0$, we take $\mathcal {H}_0 = \mathcal {C}_{t,d-1}$. Item 1 holds by definition of $\mathcal {H}_0$ and Item 2 holds by the inductive hypothesis on $\mathcal {C}_{t,d-1}$.

Now, assume that $i>0$. We will build $\mathcal {S}_i$ and $\mathcal {P}_i$ iteratively. Initialise $\mathcal {S}_i$ to $\mathcal {S}_{i-1}$ and $\mathcal {P}_i$ to $\emptyset$. For each probe $P\in \mathcal {P}_{i-1}$, let *R* be a square in a root of *P*, and add an *R*-scaled copy $\mathcal {H}_P$ of $\mathcal {H}$ in *R*. At this point, the configuration is still triangle-free as it is a disjoint union of $\mathcal {H}_{i-1}$ and copies of $\mathcal {H}$. For every probe *Q* in $\mathcal {H}_P$, we divide *Q* vertically into 4*t* rectangles of equal height with the same width as *Q*. We call these rectangles the *layers* of *Q*. Let $(L_j(Q))_{j\in \{0,\dots ,4t-1\}}$ be the layers of *Q* starting from the top.

**Fig. 2:**
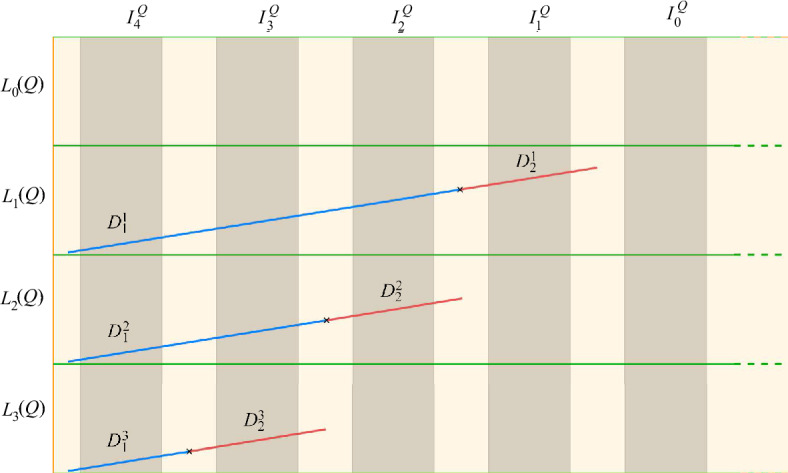
Layers of a probe *Q* drawn in green, with new segments $D_1^j$ and $D_2^j$. Example for $t=1$

First, we add the top layer $L_0(Q)$ to $\mathcal {P}_i$ as a single probe. Then, for every $j\in \{1,\dots ,4t-1\}$, we add two segments in the interior of $L_j(Q)$ to $\mathcal {S}_i$, with slope $\gamma$, that intersect at exactly one point: $D^j_1$ crossing exactly the pillars $I^Q_{j+1},\dots ,I^Q_{4t}$ horizontally, and $D^j_2$ crossing $I^Q_j$ horizontally and no other pillars. Since the slope of the diagonal of a layer is at least $\gamma$, such segments are well-defined. Moreover, the configuration is still triangle-free. On the one hand, any $D^j_1$ or $D^j_2$ intersects a set of disjoint segments over all pillars. On the other hand, any pillar, and thus any segment crossing it vertically, cannot intersect both $D^j_1$ and $D^j_2$. See Fig. 2.

**Fig. 3:**
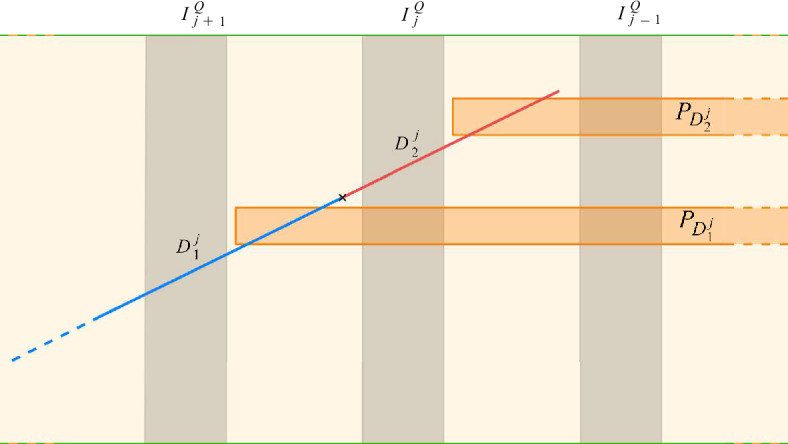
The two new probes $P_{D^j_1}$ and $P_{D^j_2}$ inside the layer $L_j(Q)$

Finally, we add to $\mathcal {P}_i$ two probes $P_{D^j_1},P_{D^j_2}$, with $P_{D^j_1}$ crossed vertically by exactly $D^j_1$ and $I^Q_0,\dots ,I^Q_j$, and $P_{D^j_2}$ crossed vertically by exactly $D^j_2$ and $I^Q_0,\dots ,I^Q_{j-1}$. See Fig. 3.

It follows from the construction that $\mathcal {P}_i$ is a set of probes for $\mathcal {S}_i$, and that every segment in $\mathcal {S}_i$ has its slope in $\Gamma \cup \{\gamma \}$ so Item 1 holds. It remains to prove that for every *t*-fold colouring $\phi$, there is a probe $P \in \mathcal {P}_i$ such that $|\phi (\mathcal {S}_i(P))| \ge 2t(d-1)+2t(1-\frac{1}{2^{i}})$. Let $\phi$ be a *t*-fold colouring of $G(\mathcal {H}_i)$. By the inductive hypothesis, there exists $P \in \mathcal {P}_{i-1}$ such that $|\phi (\mathcal {S}_{i-1}(P))| \ge 2t(d-1)+2t(1-\frac{1}{2^{i-1}})$. By Lemma 2 applied to $\mathcal {H}= \mathcal {C}_{t,d-1}^{(4t+1)}$, there exists a probe *Q* of $\mathcal {H}_P$ such that every pillar $I^Q_j$ satisfies $|\phi (\mathcal {S}_i(I^Q_j))|\ge 2t(d-1)$. For every $j \in \{0,\dots ,4t-1\}$, let $\Phi _j = \phi (\mathcal {S}_i(I^Q_j))$. Recall that the top layer $L_0(Q)$ was added as a probe, and it crosses all the $I^Q_j$s horizontally. If it contains at least $2t(d-1)+2t(1-\frac{1}{2^{i}})$ colours, then this probe satisfies Item 2 So, from now on, we may assume that *Q* contains fewer than $2t(d-1)+2t(1-\frac{1}{2^{i}})$ colours. Since $|\Phi _0| \ge 2t(d-1)$, we have $2t(d-1)+2t(1-\frac{1}{2^{i}}) - |\Phi _0| < 2t$. Therefore, at most $2t-1$ indices $j \in \{1,\dots ,4t-1\}$ are such that the set $\Phi _j$ contains a colour not in $\bigcup _{j'\le j-1} \Phi _{j'}$. Thus, for at least $4t-1-2t+1=2t$ indices $j \in \{1,\dots ,4t-1\}$, $\Phi _{j} \subseteq \bigcup _{j' < j} \Phi _{j'}$. Symmetrically, for at least 2*t* indices $j \in \{1,\dots ,4t-1\}$, we have $\Phi _{j} \subseteq \bigcup _{j' > j} \Phi _{j'}$. Thus, there is an index $j_0 \in \{1,\dots ,4t-1\}$ such that $\Phi _{j_0} \subseteq \bigcup _{j' > j_0} \Phi _{j'}$ and $\Phi _{j_0} \subseteq \bigcup _{j' < j_0} \Phi _{j'}$. Let $A = \Phi _{j_0}$, $B = \bigcup _{j' < j_0} \Phi _{j'}$ and $C = \bigcup _{j' > j_0} \Phi _{j'}$. We have $A \subseteq B$ and $A \subseteq C$. Consider the layer $L_{j_0}(Q)$ and the corresponding segments $D_1^{j_0},D_2^{j_0}$. Since $D_1^{j_0}$ and $D_2^{j_0}$ share a common point, $\phi (D_1^{j_0}) \cap \phi (D_2^{j_0}) = \emptyset$. Since $D_1^{j_0}$ crosses $I^Q_{j_0+1},\dots ,I^Q_{4t}$ horizontally, we have $\phi (D_1^{j_0}) \cap C = \emptyset$. Since $A \subseteq C$, we also have $\phi (D^{j_0}_1) \cap A = \emptyset$. Moreover, $D_2^{j_0}$ crosses $I^Q_{j_0}$ horizontally, so $\phi (D^{j_0}_2) \cap A = \emptyset$. As a result, $\phi (D^{j_0}_1)$, $\phi (D^{j_0}_2)$, and *A* are pairwise disjoint. Thus, $|\phi (D^{j_0}_1) \cup \phi (D^{j_0}_2) \cup A| = |\phi (D^{j_0}_1)| + |\phi (D_2^{j_0})| + |A| \ge t + t + 2t(d-1) = 2td$. Hence, letting $Z_{i-1} = \phi (\mathcal {S}_{i-1}(P))$, we have $|(\phi (D^{j_0}_1) \cup \phi (D^{j_0}_2) \cup A) \setminus Z_{i-1}| \ge 2td - |Z_{i-1}|$. Therefore, there is some $\alpha \in \{1,2\}$ such that $|(\phi (D^{j_0}_\alpha ) \cup A) \setminus Z_{i-1}| \ge \frac{2td-|Z_{i-1}|}{2}$. It follows that

$$ |\phi (D^{j_0}_\alpha ) \cup A \cup Z_{i-1}| \ge \frac{2td+|Z_{i-1}|}{2} \ge 2t(d-1) + 2t\left( 1-\frac{1}{2^i}\right) . $$

Then $\phi \left( \mathcal {S}_i\left( P_{D^{j_0}_\alpha }\right) \right)$ contains at least $|\phi (D^{j_0}_\alpha ) \cup B \cup Z_{i-1}| \ge |\phi (D^{j_0}_\alpha ) \cup A \cup Z_{i-1}| \ge 2t(d-1) + 2t(1-\frac{1}{2^i})$ colours. Therefore, Item 2 always holds for $\mathcal {H}_i$. This concludes the proof of the theorem. $\square$

### Corollary 4

For all positive integers *t* and *d*, there exists a multiset of segments $\mathcal {S}'_{t,d} \subseteq \mathcal {U}$ with slope number at most *d* such that $\omega (G(\mathcal {S}'_{t,d})) = 2t$ and $\chi (G(\mathcal {S}'_{t,d}))\ge 2td$.

### Proof

Let $\mathcal {C}_{t,d} = (\mathcal {S}_{t,d},\mathcal {P}_{t,d})$ be a configuration given by Theorem 3. Let $\mathcal {S}'_{t,d}$ be a *t*-blowup of $\mathcal {S}_{t,d}$, that is a configuration obtained from $\mathcal {S}_{t,d}$ by replacing every segment $v \in \mathcal {S}_{t,d}$ by *t* identical copies $v_1, \dots , v_t$. Since the graph $G(\mathcal {S}_{t,d})$ is triangle-free, we have $\omega (G(\mathcal {S}'_{t,d})) = 2t$. Let $\phi$ be a colouring of $G(\mathcal {S}'_{t,d})$. The colouring $\phi$ corresponds to a *t*-fold colouring $\phi _t$ of $G(\mathcal {S}_{t,d})$ defined by $\phi _t(v) = \{\phi (v_1), \dots , \phi (v_t)\}$ for all $v\in V(G(\mathcal {S}_{t,d}))$. By Theorem 3, there exists a probe $P\in \mathcal {P}_{t,d}$ such that $|\phi _t(\mathcal {S}_{t,d}(P))|\ge 2td$. Hence, by definition of $\phi _t$, $\phi$ uses at least 2*td* colours as claimed. $\square$

## Lower Bound for Odd $\omega$

In this section, we certify the lower bound for the $\chi$-binding function in the case of odd clique numbers. We rely on the construction of *d*-DIR graphs with $(t+1)$-fold chromatic number at least $2(t+1)d$ as guaranteed by Theorem 3. Instead of blowing up each segment by $t+1$ as in the even case, many blowups will be *t*-blowups, ensuring the clique number increases only to $2t+1$. We do so in a way that also ensures the chromatic number is $2td+1$, as desired.

### Theorem 5

For every nonnegative integer *t* and every positive integer *d*, there exists a multiset of segments $\mathcal {S}''_{t,d}$ with slope number at most *d* such that $\omega (G(\mathcal {S}''_{t,d})) = 2t+1$ and $\chi (G(\mathcal {S}''_{t,d})) \ge 2td+1$.

### Proof

Let *t* be a nonnegative integer and let *d* be a positive integer. By Theorem 3, there is a triangle-free configuration $\mathcal {C}_{t+1,d} = (\mathcal {S}_{t+1,d}, \mathcal {P}_{t+1,d})$ with slope number at most *d* such that every $(t+1)$-fold colouring of $G(\mathcal {C}_{t+1,d})$ uses at least $2(t+1)d$ colours. Let $\mathcal {S}^1, \dots , \mathcal {S}^d$ be the partition of $\mathcal {S}_{t+1,d}$ according to the slopes of the segments. For every $i \in [d]$, $G(\mathcal {S}^i)$ is a triangle-free interval graph, and so it is bipartite. Hence there is a partition $\mathcal {S}^{i,1}, \mathcal {S}^{i,2}$ of $\mathcal {S}^i$ such that the segments in $\mathcal {S}^{a,i}$ are pairwise disjoint for each $a \in \{1,2\}$. Now, let $\mathcal {S}''_{t,d}$ be obtained from $\mathcal {S}_{t+1,d}$ by blowing up every segment *v* in $\bigcup _{i \in [d-1]} \mathcal {S}^i$ and $\mathcal {S}^{d,1}$ into *t* copies $v_1, \dots , v_{t}$, and every segment *v* in $\mathcal {S}^{d,2}$ into $t+1$ copies $v_1, \dots , v_{t+1}$.

First, by construction, $\omega (G(\mathcal {S}''_{t,d})) \le 2t+1$. We now show that $\chi (G(\mathcal {S}''_{t,d})) \ge 2td+1$. Let $\phi$ be a *k*-colouring of $G(\mathcal {S}''_{t,d})$. Let us introduce new colours (1, *i*), (2, *i*), for $i \in [d]$, and suppose they are not in the image of $\phi$. For every $i \in [d-1]$ and every $v \in \mathcal {S}^{i,a}$, where $a \in \{1,2\}$, we let $\psi (v) = \{\phi (v_1), \dots ,\phi (v_t), (a,i)\}$. For every $v \in \mathcal {S}^{d,1}$, we let $\psi (v) = \{\phi (v_1), \dots , \phi (v_t), (1,d)\}$. Finally, for every $v \in \mathcal {S}^{d,2}$, we let $\psi (v) = \{\phi (v_1), \dots , \phi (v_{t+1})\}$. Note that $\psi (v)$ has size exactly $t+1$ for every $v \in \mathcal {S}_{t+1,d}$, and $\psi$ uses at most $k+2d-1$ colours (since we did not use the colour (2, *d*)). Moreover, as $\phi$ is proper and we invoke the bipartitions of the $\mathcal {S}^i$s, we deduce that $\psi$ is a $(t+1)$-fold colouring of $\mathcal {S}_{t+1,d}$. This implies that $k +2d-1 \ge 2(t+1)d$, and so $\phi$ uses $k \ge 2td+1$ colours. This proves that $\chi (G(\mathcal {S}''_{t,d})) \ge 2td+1$. $\square$

## Upper Bound for Odd $\omega$

In this section, we upper bound the $\chi$-binding function for *d*-DIR in the case of odd clique numbers, which combined with the result of the previous section settles the odd case in Theorem 1.

Let *G* be an interval graph and let $\mathcal {I}= (I_v \mid v \in V(G))$ be an interval representation of *G*. Let $\omega$ be an integer with $\omega \ge \omega (G)$. For any $x \in \mathbb {R}$, we define the set $S_x=\{u \in V(G) \mid x \in I_u\}$ of vertices corresponding to intervals containing *x*. A vertex *v* of *G* is said to be $\omega$*-uncovered* if there exists $x \in I_v$ such that $|S_x| \le \frac{\omega - 1}{2}$. Observe that $S_x$ consists of a clique of $\omega$-uncovered vertices only. We use the following lemma appearing in [7] as a tool to build colourings of *d*-DIR graphs in the case of odd $\omega$.

### Lemma 6

([7, Lem. 1]) Let *G* be an interval graph with clique number at most $\omega$. There is a colouring $\phi :V(G) \rightarrow \{0, \dots ,\omega -1\}$ of *G* such that $\phi (v)\ne 0$ for every $\omega$-uncovered vertex *v*.

### Theorem 7

For any graph *G* in *d*-DIR, if $\omega (G)$ is odd, then $\chi (G) \le d(\omega (G)-1)+1$.

### Proof

Let $(I_u \mid u \in V(G))$ be a *d*-DIR representation of *G*. Let $V_1, \dots , V_d$ be the partition of *V*(*G*) according to the slopes of the segments in its *d*-DIR representation. For every $i\in \{1,2,\dots ,d\}$, $G[V_i]$ is an interval graph and $\omega (G)\ge \omega (G[V_i])$. So by Lemma 6, there is a colouring $\phi _i$ of $G[V_i]$ using the colours $0,1,2,\dots ,\omega (G)-1$ and no $\omega (G)$-uncovered vertices of $G[V_i]$ are coloured 0. For every $i\in \{1,\dots ,d\}$ and for every $v\in V_i$, let $\phi (v)=(i,\phi _i(v))$ if $\phi _i(v) \ne 0$, and $\phi (v)=0$ otherwise. We claim that $\phi$ is a $(d(\omega (G)-1)+1)$-colouring of *G*. Indeed, if *uv* is an edge in *G* such that $\phi (u)=\phi (v)$, then $\phi (u)=\phi (v)=0$. In other words, *u* and *v* are both not $\omega (G)$-uncovered vertices and are in distinct $V_i$s. Let $i_u,i_v \in \{1,\dots ,d\}$ be such that $u \in V_{i_u}$ and $v \in V_{i_v}$. Since $\omega (G)$ is odd, the intersection point *x* of $I_u$ and $I_v$ is included in at least $\frac{\omega (G)+1}{2}$ intervals corresponding to vertices in $V_{i_u}$ and at least $\frac{\omega (G)+1}{2}$ intervals corresponding to vertices in $V_{i_v}$. This gives a clique in *G* of order $\omega (G)+1$, a contradiction. $\square$

## Concluding Remarks

Recall the following definitions. The *Hall ratio* $\rho (G)$ of a graph *G* is defined by $\rho (G)=\max _{H\subseteq G} \frac{|V(H)|}{\alpha (H)}$, where $\alpha$ denotes the independence number. The *fractional chromatic number* $\chi _f(G)$ of *G* is the infimum of *a*/*t* as $t\rightarrow \infty$ with *a* being the least integer such that *G* admits a *t*-fold *a*-colouring. Note that $\omega (G) \le \rho (G)\le \chi _f(G) \le \chi (G)$ for all *G*. Walczak [14] showed that there are triangle-free segment graphs of arbitrarily large $\rho$.

One could then ask for the same lower bounds as Theorem 1 but for $\chi _f$ or $\rho$ instead of $\chi$. While we exhibit a fixed graph in *d*-DIR with even clique number $\omega$ attaining $\chi = d \omega$, such a construction cannot be obtained for $\chi _f$ or $\rho$. Indeed, as shown in [2], already for $d=2$ and $\omega =2$, while $\chi _f$ can be arbitrarily close to 4, it must hold that $\chi _f \le 4 - \frac{1}{2^{k-1}}$, where *k* is the number of different rows in the configuration. Nevertheless, the following might hold: given *d* and even $\omega$, is there, for every $\varepsilon > 0$, a *d*-DIR graph with $\chi _f \ge d \omega - \varepsilon$ or $\rho \ge d \omega - \varepsilon$?

A possible generalisation of our problem is to consider rectangles instead of segments. In a similar manner, we can introduce the notion of the *d*-directional rectangle graphs: consider a set *d* non-parallel lines and any set of rectangles such that each of them has one of the sides parallel to one of the given lines. We want to find the $\chi$-binding function of such graphs.

This problem has been quite extensively studied in the case of axis-parallel rectangles (i.e. $d = 1$) and thanks to Chalermsook and Walczak [4] it is known that in this case $O(\omega \log \omega )$ colours always suffice; however, the lower bound remains $\Omega (\omega )$. This implies that for any *d*, no more than $O(d \omega \log \omega )$ colours are needed, and our construction from this paper can be easily adapted (by replacing the segments with “thin enough” rectangles) to show a lower bound of $\omega \cdot (d + o(1))$. The gap between linear and log-linear function remains open, though, in both $d=1$ and the general case.
